# The Association Between the Early Introduction of Solid Food and Childhood Obesity Risk: A Systematic Review

**DOI:** 10.7759/cureus.99245

**Published:** 2025-12-15

**Authors:** Surendra Gupta, Purushottam Lal, Rakesh Sharma, Abhishek Gupta, Brajesh R Chaudhary

**Affiliations:** 1 Pediatrics, Clinica Sierra Vista, Fresno, USA; 2 Family Medicine, Saint Agnes Medical Center, Fresno, USA; 3 Pediatrics, Mohawk Valley Health System, Utica, USA; 4 Child Neurology, Surya Hospital, Jaipur, IND; 5 Oral Medicine and Radiology, Chitwan Medical College, Bharatpur, NPL; 6 Pediatrics, College of Medical Sciences, Chitwan, NPL

**Keywords:** body mass index, breast feeding, childhood obesity, infant nutrition, risk factors, weaning

## Abstract

The purpose of this systematic review was to evaluate the association between the timing of solid food introduction during infancy and the risk of childhood obesity. The review specifically examined whether early introduction (before four months of age) is linked with increased risk of overweight or obesity in childhood. We conducted a systematic search of five major databases (PubMed/MEDLINE, Embase, Web of Science, Cochrane Library, and Google Scholar) from inception to January 15, 2025. Eligible studies included observational cohort, cross-sectional, quasi-experimental, and case-control studies that assessed the relationship between the timing of solid food introduction and childhood obesity outcomes. Two independent reviewers screened articles, extracted data, and assessed study quality using appropriate tools (ROBINS-I tool (Risk Of Bias In Non-randomized Studies of Interventions), AXIS, or the Newcastle-Ottawa Scale (NOS). Due to heterogeneity in study design, population, and outcome definitions, a narrative synthesis was conducted instead of meta-analysis.

Seventeen studies met the inclusion criteria out of 1,402 screened records. Most studies found that the introduction of solid foods before four months of age was associated with increased risk of childhood obesity or higher BMI z-scores. This association was more prominent in formula-fed infants. However, variation in study design, exposure classification, and measurement outcomes limited comparability. Risk of bias across studies ranged from moderate to serious, primarily due to confounding and reliance on parental recall. Early introduction of solid foods (before four months) appears to be associated with increased childhood obesity risk, particularly in the absence of breastfeeding. Nevertheless, due to variability and methodological limitations across studies, findings should be interpreted with caution. Further high-quality prospective studies using standardized outcome measures are warranted. The review protocol is registered in PROSPERO (CRD42025640375).

## Introduction and background

The global prevalence of childhood obesity has increased dramatically over the past four decades. Between 1975 and 2016, global age-standardized obesity prevalence rose from 0.7% to 5.6% in girls and from 0.9% to 7.8% in boys aged 5-19 years [1]. Although rates have plateaued in many high-income countries, they remain high, while an upward trend continues in regions such as Asia, the Middle East, North Africa, and the Caribbean. Some areas, including Polynesia, Micronesia, and the USA, report obesity rates of 20% or higher among children [1,2]. In contrast, some European countries, like Italy, and those in Southern Europe, have seen declines in obesity rates [3,4].

In the United States, the prevalence of childhood obesity has more than tripled in the last four decades, from 5% in 1978 to 18.5% in 2016 [5]. Between 1999 and 2018, obesity rates increased from 14.7% to 19.2%, and severe obesity rose from 3.9% to 6.1% [6]. Although the rate of increase has slowed since 2004, disparities remain evident, with higher prevalence among Black and Hispanic children and those from Spanish-speaking households [6]. Policy interventions, such as the 2009 revision of the WIC (Women, Infants, and Children) food package, appear to have helped reduce obesity rates among low-income children [7]. Despite this, overall prevalence remains high, and early childhood is considered a critical window for intervention [5].

The rise in childhood obesity poses serious public health concerns. It is linked to an increased risk of developing chronic conditions such as type 2 diabetes, cardiovascular disease, hypertension, fatty liver disease, and certain cancers, which can persist into adulthood [2,8,9]. Psychological impacts, including depression, low self-esteem, and social exclusion, are also common in children with obesity [10]. Moreover, children with obesity are more likely to remain obese as adults, perpetuating cycles of poor health and increased healthcare use [2,9].

Economically, childhood obesity drives rising healthcare expenditures by increasing the need to manage both short- and long-term complications, and it also diminishes workforce productivity through earlier disability and chronic disease [2,11]. Preventing childhood obesity through early and sustained interventions is more cost-effective than treatment, and should prioritize breastfeeding promotion, healthy feeding practices, and increasing physical activity [2,9,12]. Multicomponent strategies involving families, schools, and policy measures such as food labeling, sugar taxes, and restrictions on marketing unhealthy foods are necessary to address the complex socio-environmental contributors to childhood obesity [12-14].

Major health organizations offer specific guidance on the timing of complementary feeding. The World Health Organization (WHO, 2023) advises exclusive breastfeeding for the first six months of life, after which solid foods should be introduced alongside continued breastfeeding [15]. Similarly, the American Academy of Pediatrics (AAP, 2022) advises exclusive breastfeeding for about six months, after which complementary foods should be introduced alongside continued breastfeeding [16]. The European Society for Paediatric Gastroenterology, Hepatology, and Nutrition (ESPGHAN, 2017) recommends introducing complementary foods between four and six months of age, ideally around six months, in combination with breastfeeding [17].

Complementary feeding refers to the gradual process of introducing solid or semi-solid foods to an infant’s diet alongside continued breastfeeding or formula feeding, typically beginning between four and six months of age. Responsive feeding is a caregiver-led approach that involves recognizing and responding appropriately to an infant’s hunger and satiety cues, thereby supporting the child’s ability to self-regulate food intake. Providing these definitions helps clarify key concepts and improves accessibility for readers who may be less familiar with pediatric nutrition terminology.

Recent studies support these recommendations. For instance, introducing fruits and vegetables between five and eight months of age, rather than before five months, has been linked to a reduced risk of obesity at seven to nine years [10]. The hypothesis that early introduction of solid food may contribute to later obesity is grounded in concerns about disrupted energy balance and altered metabolic programming. According to the "early protein hypothesis," the early introduction of solids or formula, often higher in protein than breast milk, may accelerate weight gain and predispose infants to obesity by affecting metabolic pathways [12]. Additionally, the timing and type of complementary foods influence the development of self-regulation in energy intake. Early introduction may interfere with feeding behaviors and the child’s ability to recognize internal hunger and satiety cues, thereby increasing the risk of obesity [10].

Evidence also shows that formula-fed infants are more likely to experience rapid weight gain compared to breastfed infants, potentially due to higher protein content and less responsive feeding practices [18-20]. Breastfeeding, in contrast, has been consistently associated with a modest reduction in obesity risk, even after adjusting for various confounders [21,22]. Responsive feeding interventions that encourage parents to feed their children based on hunger and satiety cues, while delaying solid food introduction, can improve feeding behaviors and potentially reduce the risk of obesity [10,23].

Despite the extensive research on infant feeding and obesity, the existing literature presents several important limitations that hinder clear interpretation and informed policy guidance. One major issue is the inconsistency in defining “early” and “late” introduction of solid foods. Studies have employed varying age cutoffs such as before four months, between four to five months, or after six months, making it difficult to compare results across studies and synthesize findings effectively [24-27]. In addition to inconsistent definitions, the outcomes measured across studies exhibit significant variability. While some focus on BMI percentiles or weight-for-age z-scores, others evaluate the prevalence of overweight and obesity or use broader markers such as fat distribution and growth patterns [19,28]. This diversity in outcome measures contributes to the inconsistent conclusions reported in the literature.

Methodologically, many studies rely on observational data, which introduces potential for recall bias and confounding. Common confounders such as maternal BMI, education level, and breastfeeding duration are often inconsistently controlled for, further weakening the strength of the evidence [24,26,29,30]. Moreover, some studies report associations between early solid food introduction and obesity only in specific subgroups, such as formula-fed infants, while others find no significant relationships at all [24,25,27,31]. Given these inconsistencies, there is a clear need for an updated systematic review that applies standardized definitions, specifically comparing introduction before versus at or after four months, and consistent, validated obesity-related outcomes, such as BMI percentiles and standardized overweight categories. Additionally, robust synthesis should account for key confounding variables and examine subgroup differences, particularly between breastfed and formula-fed infants [24-27].

This systematic review aims to evaluate how the early introduction of solid foods, defined as before four months of age, compared to later introduction at or after four months, influences the risk of childhood obesity among infants and young children aged zero to five years. The review will focus on both primary and secondary outcomes, with childhood obesity (BMI ≥95th percentile) serving as the primary outcome, and additional outcomes including overweight risk, growth trajectories, feeding behaviors, and potential adverse effects. The study will include observational designs such as cohort, case-control, and cross-sectional studies as well as randomized controlled trials (RCTs). The primary aim is to systematically assess the association between early solid food introduction and the risk of developing childhood obesity. Secondary objectives include examining variations in results across different populations, study designs, and settings, and evaluating consistency in findings. The research tasks involve identifying and screening relevant studies, extracting and synthesizing outcome data related to solid food introduction timing, critically assessing study quality and risk of bias, and interpreting findings in the context of current public health recommendations and policy implications.

## Review

Material and methods

Protocol and Registration

This systematic review was conducted in accordance with the PRISMA (Preferred Reporting Items for Systematic Reviews and Meta-Analyses) 2020 Statement, ensuring transparency and methodological rigor throughout the review process [32]. The review protocol was prospectively registered with PROSPERO under the registration number CRD42025640375 to enhance reproducibility and minimize reporting bias. A completed PRISMA checklist and flow diagram are provided as supplementary materials to document the study selection process and adherence to reporting standards [32]. The review was guided by the following focus question, developed using the PICO (Patient/Problem, Intervention, Comparison, and Outcome) framework: how does the early introduction of solid foods (before four months of age) compared to later introduction (at or after four months) influence the risk of childhood obesity in infants and young children?

Sources of Literature and Search Methodology

The literature search for this review was performed across five major bibliographic databases: Embase, MEDLINE (via PubMed), Web of Science Core Collection, Cochrane Library, and Google Scholar. To ensure comprehensive coverage, additional sources were identified through manual screening of reference lists from included studies and relevant systematic reviews, as well as searches of clinical trial registries, including ClinicalTrials.gov and the WHO International Clinical Trials Registry Platform. The search encompassed publications from each database’s inception through January 15, 2025, and was rerun before the final analysis to capture the most recent studies.

Only peer-reviewed scientific publications, including journal articles, dissertations, monographs, and textbooks available in either electronic or print format, were included, while non-peer-reviewed materials were excluded. Gray literature from Google Scholar was considered to minimize publication bias. The search strategy utilized a combination of controlled vocabulary and free-text keywords: ("early introduction" OR "complementary feeding" OR "solid food introduction") AND ("childhood obesity" OR "pediatric obesity" OR "BMI" OR "overweight" OR "infant growth"). For example, the full PubMed search string was: ("Infant"[MeSH] OR infant OR baby OR babies) AND ("Feeding Behavior"[MeSH] OR "complementary feeding" OR "solid food introduction") AND ("Obesity"[MeSH] OR obesity OR overweight OR "body mass index" OR BMI). Search strings were adapted for syntax differences across databases, and filters for English language and human studies were applied.

Eligibility and Inclusion Criteria

Studies were considered eligible for inclusion if they met the following criteria: the population consisted of infants and young children aged zero to five years, excluding preterm infants and those with medical conditions affecting feeding or growth; the exposure involved the introduction of solid or semi-solid foods before four months of age; the comparator was the introduction of solids at or after four months of age; and the outcomes included indicators of childhood obesity such as BMI ≥95th percentile, overweight prevalence, and growth z-scores, along with secondary outcomes including growth trajectories and potential adverse effects. Eligible study designs encompassed RCTs, cohort studies, case-control studies, and cross-sectional studies. Each study was rigorously evaluated for eligibility based on its alignment with the predefined PICO framework and its adherence to methodological quality and relevance to the review protocol.

Study Selection and Screening Process

All records were imported into systematic review management software (Rayyan) [33]. Two independent reviewers screened titles and abstracts for inclusion, followed by full-text assessment of potentially eligible studies. Reviewers were blinded to each other’s decisions, and any disagreements were resolved by discussion or consultation with a third reviewer.

**Figure 1:**
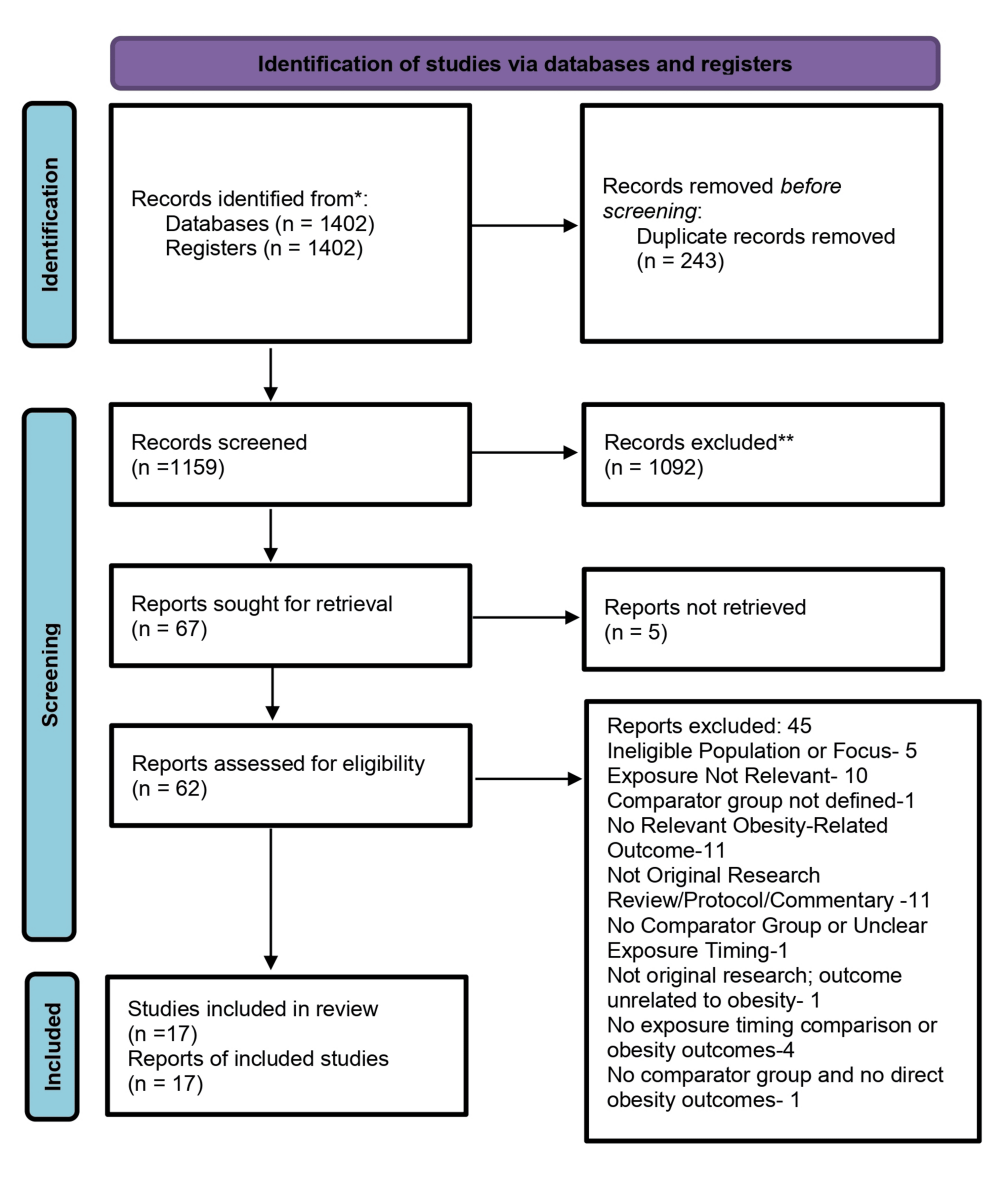
PRISMA 2020* flow diagram depicting the study selection process ^*^[32] PRISMA: Preferred Reporting Items for Systematic Reviews and Meta-Analyses

To evaluate consistency between reviewers during study selection, we employed a dual, independent screening process for titles and abstracts, as well as full texts. Although Cohen’s Kappa (κ) statistic was not originally included in the study protocol, the reviewer’s recommendation is acknowledged. Future updates of this review will incorporate the calculation of κ to formally quantify interobserver agreement. Any disagreements that arose during screening in the present review were resolved through discussion and, when necessary, consultation with a third reviewer, thereby ensuring methodological rigor and minimizing selection bias. The PRISMA 2020 flow diagram illustrating the study selection process is shown in Figure 1.

Data Extraction and Quality Assessment

Data extraction was conducted independently by two reviewers using a standardized extraction form. Extracted variables included study characteristics (title, year, country, funding), design type, participant demographics, exposure and comparator details, outcome measures, and statistical estimates (risk ratio (RR), odds ratio (OR), hazard ratio (HR), mean difference (MD), 95% confidence interval (CI). Missing data were requested by contacting study authors directly, and all correspondence was documented.

Risk of Bias Assessment

Methodological quality and risk of bias were independently assessed by two reviewers using validated tools appropriate for each study design. The ROBINS-I tool (Risk Of Bias In Non-randomized Studies of Interventions) was employed to evaluate quasi-experimental studies, prospective cohort studies, and longitudinal cohort studies [34]. For cross-sectional studies utilizing large national or multinational datasets, the AXIS tool was applied [35]. In contrast, the Newcastle-Ottawa Scale (NOS) was used to assess matched case-control studies nested within cohort studies [36]. Each study underwent evaluation for risk of bias at both the study and outcome levels and was categorized as having low, moderate, or high risk of bias based on predefined criteria. These quality ratings informed the weighting of studies in the synthesis and were incorporated into sensitivity analyses to examine the robustness and reliability of the review’s findings.

Risk of Bias Across Studies

To evaluate potential biases affecting the overall body of evidence, formal assessments of publication bias and selective reporting were conducted. For each outcome with 10 or more studies included in the meta-analysis, funnel plots were generated to visually assess asymmetry, which could suggest the presence of publication bias. Additionally, Egger’s test was performed to statistically evaluate funnel plot asymmetry and detect small-study effects. Potential selective outcome reporting within individual studies was also examined during the risk of bias assessment, particularly in cases where pre-specified outcomes from study protocols (when available) did not align with the reported results. Studies identified as having a high risk of selective reporting were flagged and accounted for in sensitivity analyses to evaluate the robustness of pooled effect estimates. The findings from these bias assessments were used to guide the interpretation of results and to inform the overall confidence in the synthesized evidence.

Data Synthesis and Statistical Methods

Given the methodological heterogeneity across included studies, particularly in study design, population characteristics, exposure definitions, and outcome measures, a meta-analysis was not feasible. Instead, a structured narrative synthesis was undertaken to summarize findings. Data were organized thematically by timing of solid food introduction (before four months, four to six months, ≥6 months) and stratified where possible by breastfeeding status and study design. We grouped outcomes by measurement type, such as BMI percentiles, weight-for-age z-scores, and prevalence of overweight or obesity. Pre-specified subgroup themes, including infant feeding method, timing of obesity assessment, and study quality, were explored descriptively. Tabular synthesis and graphical summaries (e.g., risk-of-bias traffic light plots) were used to aid comparison across studies. All data extraction steps, subgroup classification, and risk-of-bias assessments were performed in duplicate to improve transparency and reproducibility. The risk of publication bias and small-study effects could not be assessed quantitatively due to the insufficient number of comparable studies that reported pooled effect estimates.

A meta-analysis was not conducted due to substantial heterogeneity across studies in terms of population characteristics, definitions of early solid food introduction, outcome measures (e.g., BMI z-scores, overweight prevalence), timing of outcome assessment, and covariates adjusted in the statistical models. These variations limited the feasibility of generating pooled effect estimates. In accordance with the SWiM (Synthesis Without Meta-analysis) guidelines, studies were grouped thematically based on outcome type (e.g., overweight prevalence, BMI percentiles) and exposure timing (before four months, four to six months, ≥6 months). Where applicable, subgroup findings (e.g., based on breastfeeding status or socioeconomic strata) were descriptively synthesized and reported in supplementary tables to facilitate transparency and comparability. No funnel plots or Egger’s test were applied due to the absence of meta-analysis. Descriptive statistics, reported p-values, ORs, RRs, and CIs were extracted from primary studies and presented in the synthesis tables. No further statistical testing or regression analyses were conducted by the review team.

Results

Study Selection

A total of 1,402 records were identified through systematic searches across selected databases. Using Rayyan, 335 records were identified as suspected duplicates, and 243 were subsequently confirmed, resulting in 1,159 unique records for title and abstract screening [33]. Two independent reviewers screened all 1,159 articles. After applying the eligibility criteria, 67 records were selected for full-text review. Of these, five full-texts could not be retrieved, and 45 articles were excluded (Table 1) after full-text assessment due to reasons such as inappropriate study design, incorrect population, or insufficient outcome data.

**Table 1:** Summary of excluded studies examining the association between timing of solid food introduction and childhood obesity risk

S. no	Title	Author and year	Reason for exclusion	Explanation
1	Adherence with early infant feeding and complementary feeding guidelines in the Cork BASELINE Birth Cohort Study [37]	O’Donovan et al., 2015	No relevant outcome	The study focuses on feeding guideline adherence, not on obesity or weight-related outcomes
2	Age at weaning and infant growth: primary analysis and systematic review [38]	Vail et al., 2015	No relevant outcome	Analyzes weight gain in infancy but does not assess obesity, overweight, or BMI outcomes
3	Association of infant child care with infant feeding practices and weight gain among US infants [39]	Kim and Peterson, 2008	Exposure not relevant	Focuses on child care and general feeding practices, not specifically on timing of solid foods
4	Associations between dietary intake before 6 months of age and rapid weight gain among HIV-exposed uninfected infants [40]	Neri et al., 2017	Ineligible population	Study focused on HIV-exposed infants, which are excluded per your review criteria
5	Associations between early introduction to complementary foods, subsequent cereal-added bottle feeding and daily macronutrient intake among infants [41]	Dharod et al., 2023	No relevant outcome	Investigated feeding and nutrition patterns, but did not assess obesity, weight, or BMI outcomes
6	Associations of infant feeding and timing of linear growth and relative weight gain during early life with childhood body composition [42]	De Beer et al., 2015	Exposure not isolated	Did not isolate timing of solid food introduction (<4 months vs. ≥4 months) as a distinct variable
7	Associations of infant feeding practices with abdominal and hepatic fat measures in childhood in the longitudinal Healthy Start Study [28]	Cohen et al., 2024	Exposure not isolated	Did not isolate timing of solids; exposure combines mode and timing, limiting causal interpretation
8	Complementary feeding, infant growth, and obesity risk: timing, composition, and mode of feeding [43]	Grote et al., 2018	Narrative review with analysis of cohort data from the prospective European Childhood Obesity Project (CHOP)	1,000 healthy, singleton infants from 5 European countries. Recruited between birth and 8 weeks of age and followed for 2 years
9	Timing of complementary feeding introduction and adiposity throughout childhood [44]	Gingras et al., 2019	Outcome measured outside the target age range	The study primarily evaluates adiposity outcomes in midchildhood (mean age ~7.9 years) and early adolescence (mean age ~13.2 years), which exceeds the 0–5 year target range specified in the screening criteria
10	Body mass index, adiposity rebound and early feeding in a longitudinal cohort (Raine Study) [45]	Chivers et al., 2010	Outcome measured outside the target age range	Although early feeding was assessed, the outcomes (BMI, adiposity rebound) were primarily measured beyond 5 years, including at 8, 10, and 14 years, which violates the outcome age criteria of 0–5 years
11	Bottle-weaning intervention and toddler overweight [46]	Bonuck et al., 2014	Exposure does not match the review criteria	The study investigates the impact of a bottle-weaning intervention (i.e., reducing prolonged bottle use) rather than the timing of solid food introduction, which is the specified exposure in your review criteria
12	Can optimal complementary feeding improve later health and development? [47]	Fewtrell, 2016	Not an original study (review or commentary)	This article is a narrative review that summarizes findings from other studies and systematic reviews. It does not report original data or conduct primary research, which disqualifies it per the inclusion criteria for original studies only
13	Diet and growth in infancy: relationship to socioeconomic background and to health and development in the Avon Longitudinal Study of Parents and Children [48]	Emmett and Jones, 2014	Exposure and outcomes not clearly aligned with review criteria	While the paper discusses infant diet and growth, it does not clearly focus on the timing of solid food introduction before 4 months as a primary exposure, nor does it provide childhood obesity-specific outcomes (0–5 years) in the context required by the review
14	Differences in infant feeding practices between Chinese-born and Australian-born mothers living in Australia: a cross-sectional study [49]	Bolton et al., 2018	Outcome not relevant to childhood obesity	Although the study addresses the timing of complementary feeding, it does not evaluate obesity-related outcomes (e.g., BMI, body fat, weight gain) in children aged 0–5 years, making it ineligible for inclusion
15	Differences in weaning practice, food and nutrient intake between breast- and formula-fed 4-month-old infants in England [50]	Noble and Emmett, 2006	No relevant obesity-related outcomes	While this study focuses on timing and type of complementary feeding, it does not assess childhood obesity, BMI, weight gain, or body composition outcomes, and is therefore excluded based on your outcome criteria
16	Does a baby-led approach to complementary feeding alter the risk of choking and growth faltering in infants aged 0–12 months? [51]	Fangupo, 2016	Outcome not related to obesity	The study examines choking and growth faltering risks associated with baby-led weaning but does not evaluate obesity-related outcomes such as BMI, body fat, or weight gain, which are required for inclusion
17	Evaluation of a feasibility study addressing risk factors for childhood obesity through home visits [52]	Wen et al., 2009	No comparator group; not designed for exposure-effect assessment	This pilot study explored feasibility rather than evaluating early solid food introduction (<4 months) as an exposure with a comparator group. It lacked outcome analysis linking timing of solids to childhood obesity, thus it does not meet core inclusion criteria
18	Infant feeding in relation to eating patterns in the second year of life and weight status in the fourth year [53]	Abraham et al., 2012	Timing of solid food introduction not clearly defined	Although the study assesses early feeding patterns and later weight status, it does not explicitly examine the timing of complementary feeding before 4 months or use clear exposure/comparator groups based on timing, which is required by your criteria
19	Infant feeding practices and body mass index up to 7.5 years in the French nationwide ELFE study [54]	Camier et al., 2024	Outcome age exceeds inclusion range	Although the study includes BMI outcomes, the main analysis extends to 7.5 years of age, which exceeds the target outcome window of 0–5 years defined in the inclusion criteria
20	Modifiable risk factors in the first 1000 days for subsequent risk of childhood overweight in an Asian cohort: significance of parental overweight status [55]	Aris et al., 2018	Timing of solid food introduction not clearly defined	Although the study examines several risk factors for early childhood obesity, it does not specifically focus on the timing of solid food introduction before 4 months, which is a required exposure criterion for your systematic review
21	Mother, infant, and household factors associated with the type of food infants receive in developing countries [56]	Yarnoff et al., 2014	Focus on food type, not timing	The study investigates types of complementary foods given to infants in various countries, but does not assess timing of solid food introduction or its association with obesity, thus does not meet the key exposure and outcome criteria
22	Nutritional implications of baby-led weaning and baby food pouches as novel methods of infant feeding: protocol for an observational study [57]	Taylor et al., 2021	Study protocol (not original results)	This document is a study protocol, outlining the design of future research without presenting any original data or results, and therefore does not meet the inclusion criteria for original studies
23	Nutritional profile of commercial infant and toddler food products available in Klang Valley [58]	Razak and Muniandy, 2019	No obesity-related outcomes measured	The study analyzes the nutrient content (e.g., sugar, sodium, fat) of commercial infant/toddler food products, but does not assess associations between timing of food introduction and childhood obesity, nor does it report any child health outcomes like BMI or weight
24	Nutritional requirements of infants and need for supplementing milk diet with infant weaning foods [59]	Narain and Dubash, 1976	Not original research (narrative review/opinion)	The article provides general recommendations and expert opinions on the timing and necessity of weaning foods, but does not report original data or assess childhood obesity-related outcomes, which excludes it based on your criteria
25	Promoting weaning practices and growth of Egyptian infants by using communication for behavioral development approach [60]	Metwally et al., 2022	Exposure timing unclear; outcome not obesity-focused	Although the study promotes improved weaning practices and growth, it does not clearly specify timing of solid food introduction before 4 months or measure obesity-specific outcomes like BMI or body fat in the 0–5 year range
26	Prospective associations of infant food exposures and appetitive traits with early childhood diet quality [61]	Nansel et al., 2024	Outcome unrelated to obesity	The study investigates associations between infant feeding practices and later diet quality, not obesity-related outcomes such as BMI, weight gain, or adiposity in children aged 0–5 years; thus, it does not meet the outcome criteria
27	Protein and growth during the first year of life: a systematic review and meta-analysis [62]	Milani et al., 2023	Study type excluded (systematic review	This article is a systematic review and meta-analysis, not an original research study, and therefore does not meet the inclusion criteria requiring original data
28	Protein intake during the period of complementary feeding and early childhood and the association with body mass index and percentage body fat at 7 y of age [63]	Günther et al., 2007	Outcome age exceeds inclusion range	Although the study assesses dietary intake during complementary feeding and obesity-related outcomes, these outcomes are measured at 7 years of age, which exceeds the 0–5 year age range specified in your criteria
29	Protocol for a cluster randomised trial evaluating a multifaceted intervention starting preconceptionally-Early Interventions to Support Trajectories for Healthy Life in India (EINSTEIN): a Healthy Life Trajectories Initiative (HeLTI) Study [64]	Kumaran et al., 2021	Study protocol, not original results	This publication is a study protocol describing the planned design for a future intervention and does not report original outcome data, which excludes it from your systematic review based on study type criteria
30	Relations between high ponderal index at birth, feeding practices and body mass index in infancy [65]	Lande et al., 2005	Timing of solid food introduction not clearly defined	Although the study evaluates infant BMI and feeding practices, it does not specify whether solids were introduced before 4 months, nor does it clearly define exposure and comparator groups based on timing of introduction as required by your criteria
31	Revised infant dietary recommendations: the impact of maternal education and other parental factors on adherence rates in Iceland [66]	Thorisdottir et al., 2013	Outcome unrelated to obesity	The study investigates adherence to dietary guidelines, maternal education, and cow’s milk consumption, but does not measure obesity-related outcomes such as BMI, overweight status, or adiposity in children 0–5 years of age
32	S3-Guideline on allergy prevention: 2014 update: guideline of the German Society for Allergology and Clinical Immunology (DGAKI) and the German Society for Pediatric and Adolescent Medicine (DGKJ) [67]	Schäfer et al., 2014	Not original research; outcome unrelated to obesit	This is a clinical guideline focused on allergy prevention, not an original research article. It also does not address timing of solid food introduction in relation to childhood obesity, and is therefore excluded on both study type and outcome grounds
33	Savoring sweet: sugars in infant and toddler feeding [68]	Murray, 2017	Not original research; outcome unrelated to obesity	This article is a narrative review exploring sugar exposure and taste development in infants and toddlers. It does not report original data, nor does it investigate the timing of solid food introduction or measure obesity-related outcomes, and thus does not meet the inclusion criteria
34	Science base of complementary feeding practice in infancy [69]	Michaelsen et al., 2010	Not original research (narrative review)	Summary of current knowledge and policy without presenting original data or specific analysis of timing vs. obesity outcomes
35	Socioeconomic status, infant feeding practices and early childhood obesity [70]	Gibbs and Forste, 2014	Comparator group not defined for timing of solid food introduction	While the study investigates early introduction of solids (<4 months), it does not clearly define or analyze a comparator group introduced to solids at ≥4 months of age
36	The Baby Bites Text Messaging Project with randomized controlled trial: texting to improve infant feeding practices [71]	Davis et al., 2023	Focus is on feeding practices, not timing of solid food introduction	The study aims to improve infant feeding behaviors through text messaging but does not evaluate the timing of solid food introduction or compare before vs. after 4 months
37	The Early Prevention of Obesity in CHildren (EPOCH) Collaboration--an individual patient data prospective meta-analysis [72]	Askie et al., 2010	Not a single primary study	This is a protocol for a prospective meta-analysis combining data from multiple RCTs. It is not an individual RCT but a collaborative analytic project, so it cannot be assessed for inclusion as a standalone study
38	The NOURISH randomised control trial: Positive feeding practices and food preferences in early childhood - a primary prevention program for childhood obesity [73]	Daniels et al., 2009	Exposure timing does not meet criteria	Although the intervention starts between 4 and 7 months, it does not assess solid food introduction before 4 months, which is the exposure required by your criteria
39	Time trends and social inequalities in infant and young child feeding practices: national estimates from Brazil's Food and Nutrition Surveillance System, 2008-2019 [74]	De Souza et al., 2023	No exposure timing comparison or obesity outcomes	The study describes feeding practice trends and inequalities over time but does not compare early (<4 months) vs later (≥4 months) introduction of solids or report childhood obesity outcomes
40	Timing and pattern of growth faltering in children up-to 18 months of age and the associated feeding practices in an urban setting of Sri Lanka [75]	Sithamparapillai et al., 2022	No comparator group and no direct obesity outcomes	While the study explores feeding practices and growth patterns, it does not compare early (<4 months) vs later solid food introduction or assess obesity-related outcomes (e.g., BMI, overweight)
41	Timing of allergenic food introduction to the infant diet and risk of allergic or autoimmune disease: a systematic review and meta-analysis [76]	Ierodiakonou et al., 2016	Outcomes not related to obesity	This study investigates the relationship between timing of allergenic food introduction and risk of allergy or autoimmune disease, not obesity-related outcomes
42	Timing of complementary feeding and associations with maternal and infant characteristics: a Norwegian cross-sectional study [77]	Helle et al., 2018	No obesity-related outcomes assessed	The study explores associations between complementary feeding timing and maternal/infant characteristics, but does not assess obesity, BMI, or weight gain in children
43	Timing of complementary feeding and infant growth trajectories in prospective cohort studies: a systematized review and analysis of socioecological variation [78]	Martin and Glass, 2025	Study type: review	This is a systematized review, not an original empirical study. Your criteria exclude reviews, protocols, and editorials from inclusion
44	To feed or let eat! A scale of independence, exploration, and family to measure baby-led weaning as a complementary feeding approach [79]	Studer‐Perez et al., 2023	Does not measure timing or obesity outcomes	This study focuses on the development and validation of a scale to measure baby-led weaning behaviors. It does not assess timing of solid food introduction or childhood obesity-related outcomes
45	Validation of selected 2021 infant and young child feeding indicators for appropriate complementary feeding in relation to dietary adequacy and anthropometric status [80]	Goyena et al., 2023	No clear timing of solid food introduction	Evaluates complementary feeding indicators and their relationship to diet quality and anthropometrics, but does not analyze timing of introduction in relation to obesity risk

After manual verification, 88 duplicate records were removed, and four were retained for further review. Ultimately, 17 studies met all inclusion criteria and were included in the final assessment (Table 2).

**Table 2:** Summary of included studies examining the association between timing of solid food introduction and childhood obesity risk

Study design/author and date	Population	Intervention	Comparator	Outcomes measured	Key findings	Limitations
Prospective cohort study (Finnish cohort of healthy full-term infants). (Differding et al., 2020) [81]	Followed from birth to 5 years	Timing of complementary feeding (solid food introduction): before 4 months ≥4 months Breastfeeding duration	Reference: Infants introduced to solids at ≥4 months Breastfeeding duration stratified (shorter vs. longer)	BMI at age 5 Gut microbiota composition at age 5 (via stool sample sequencing)	Early complementary feeding (before 4 months) combined with shorter breastfeeding duration was associated with higher BMI at age 5. No significant association with gut microbiota composition. Suggests interaction between feeding timing and breastfeeding duration affects obesity risk.	BMI assessed only at age 5, limiting insight into earlier growth patterns. Small sample size may reduce statistical power. Microbiota findings inconclusive due to single time point and potential confounding. Generalizability may be limited to Nordic populations
Secondary data analysis of a prospective cohort (Infant Feeding Practices Study II). (Gaffney et al., 2012) [82]	U.S. mothers and infants from birth to 12 months. Nationally distributed sample from the Infant Feeding Practices Study II	Timing of solid food introduction (before 4 months, 4–6 months, ≥6 months). Other feeding behaviors: juice consumption, breastfeeding intensity, bottle-to-bed practices	Infants introduced to solid foods at ≥6 months. Infants with low vs. medium/high breastfeeding intensity. Juice intake frequency (none/low vs. frequent)	Weight-for-age z-score (WAZ) at 12 months	Early solid food introduction (before 4 months) associated with higher WAZ at 12 months. Low breastfeeding intensity associated with higher WAZ. Frequent juice consumption linked to higher WAZ. Bottle-to-bed practice not significantly associated with WAZ	Self-reported data may introduce recall and reporting bias. Residual confounding possible despite multivariate adjustment. Generalizability limited due to voluntary sample from IFPS II cohort
Nationally representative prospective cohort study (Millennium Cohort Study – UK). (Griffiths et al., 2009) [83]	From birth to 3 years of age.	Infant feeding practices: Breastfeeding initiation. Duration of breastfeeding (before 4 months vs. ≥4 months). Age at introduction of solids (before 4 months vs. ≥4 months)	Infants who were not breastfed or were breastfed for less than 4 months. Infants introduced to solids before 4 months	Conditional weight gain z-scores from birth to 3 years (adjusted for birth weight, age, sex, and confounding factors)	Infants who were not breastfed or breastfed for before 4 months had significantly higher weight gain z-scores compared to those breastfed for ≥4 months. Early introduction of solids (before 4 months) was initially associated with increased weight gain but became non-significant after adjusting for child height. Prolonged breastfeeding may be protective against rapid weight gain in early childhood.	Retrospective recall of infant feeding at 9 months may introduce recall bias. Exclusion of ethnic minority children may affect generalizability. Residual confounding and reverse causation (e.g., mothers stopping breastfeeding due to child’s high weight gain) are possible
Longitudinal cohort study using data from the Infant Feeding Practices Study II (IFPS II). (Horodynski et al., 2017) [84]	Followed from birth through 12 months	Age at complementary food introduction (measured monthly): before 4 months, 4–5 months, ≥6 months Breastfeeding duration	Reference group: Infants introduced to complementary foods at 4–5 months. Breastfed vs. non-breastfed infants	BMI-for-age z-score (BMIZ) growth velocity from birth to 12 months. Assessed monthly using mixed-effects growth curve modeling	Introduction of complementary foods before 4 months was significantly associated with increased BMIZ growth velocity compared to introduction at 4–5 months. Longer breastfeeding duration was independently associated with slower BMIZ velocity. Findings remained robust after controlling for socioeconomic and maternal variables	Self-reported feeding practices may introduce recall or reporting bias. Study limited to first year of life – no long-term obesity outcomes. Generalizability limited to U.S. population represented by IFPS II (higher education, income)
Cross-sectional study using data from the U.S. Special Supplemental Nutrition Program for Women, Infants, and Children (WIC). (Huh et al., 2011) [27]	Age at outcome: 2 to 5 years	Age of introduction of solid foods: before 4 months, 4–6 months (reference group), 6 months	Infants introduced to solids between 4–6 months (reference group)	Obesity at ages 2–5 years, defined as BMI ≥95th percentile for age and sex	Introduction of solids before 4 months was significantly associated with increased odds of obesity in preschool age. Association was particularly strong among children never breastfed or breastfed before 4 months	Cross-sectional design prevents causal inference. Feeding practices were self-reported, introducing potential recall bias. Sample may not be generalizable beyond low-income WIC population
Quasi-Experimental Study. (Jiang et al., 2019) [85]	Infants followed from birth to 24 months in Shanghai, China	Weekly SMS messages on infant feeding from third trimester to 12 months postpartum	Mothers in the control group received routine maternal and child health care without the SMS messages	BMI, BMI z-score, and weight-for-length z-score at 12 and 24 months; introduction of solids before 4 months	No significant difference in BMI or z-scores between groups. Introduction of solid foods before 4 months was significantly associated with higher BMI, BMI z-score, and weight-for-length z-score at 24 months	Non-randomized design; differences in maternal characteristics between groups; SMS intensity may be too low; cost-effectiveness not evaluated; missing data on physical activity and screen time
Cross-sectional study with matched case-control sub-sample. (Jingxiong et al., 2009) [86]	4,654 children aged 1–35 months from urban Beijing	Feeding practices: Duration of breastfeeding. Use of formula during first 4 months. Introduction of semi-solid foods before 4 months. Energy intake assessed in 12–35 month olds	Children not exposed to early formula feeding or early semi-solid food introduction. Breastfed for ≥4 months vs. before 4 months. Normal-weight matched controls	Prevalence of overweight (defined as weight-for-length/height ≥2 SD above WHO median)	Overweight children: Less likely to be breastfed for ≥4 months, more likely to receive formula or semi-solid foods before 4 months, had higher total energy intake; parental overweight and lower education levels were significantly associated with child overweight	Cross-sectional design limits ability to infer causality. Dietary intake based on 24-hour recall may have recall bias.Cultural context may limit generalizability to other populations
Cross-sectional study using pooled data from multiple European cohorts. (Moschonis et al., 2017) [87]	Preschool-aged children (mostly aged 2 to 5 years) from 4 European countries	Timing of solid food introduction. Duration of breastfeeding. Feeding type during infancy (exclusive breastfeeding vs. formula)	Breastfed vs. formula-fed. Early (before 4 months) vs. later (≥4 months) introduction of solids	BMI and obesity prevalence at preschool age (2–5 years). Weight-for-age and height-for-age indices	Children who were breastfed longer and introduced to solids after 4 months had lower rates of overweight and obesity. Early formula feeding and early solids were associated with higher BMI and weight-for-age z-scores	Cross-sectional design: no causality inferred. Feeding practices based on parental recall. Combined cohorts may have variable data collection methods. Outcomes in UK schoolchildren and adolescents are not relevant to this review, but preschool data are valid
Cross-sectional study using data from the U.S. Early Childhood Longitudinal Study—Birth Cohort (ECLS-B). (Moss and Yeaton, 2014) [88]	Weight status measured at 2 years of age	Timing of solid food introduction (before or after 4 months). Duration of breastfeeding	Children introduced to solids after 4 months. Comparison between exclusively breastfed vs. non-exclusively breastfed infants	Weight status at 2 years (categorized as healthy weight vs. obese) based on BMI percentiles	Delayed solid food introduction (≥4 months) and longer breastfeeding duration were both associated with lower odds of obesity at age 2. Children introduced to solids before 4 months had significantly higher obesity risk (OR = 1.40)	Cross-sectional design limits causal inference. Feeding practices were self-reported, possibly introducing recall bias. Limited control for post-infancy lifestyle and diet factors. Potential residual confounding despite adjustments
Prospective birth cohort study (ALSPAC – Avon Longitudinal Study of Parents and Children). (Ong et al., 2006) [89]	Data collected at 4 months of age and followed up to 5 years	Timing of solid food introduction: <2 months, 2–3 months, ≥4 months	Comparison by feeding type and age of solid food introduction. Breastfed vs. formula/mixed-fed infants. Early (<2 months) vs. later (>4 months) solid food introduction	Weight gain from birth to 1, 2, and 3 years, BMI at ages 1 to 5 years, risk of overweight/obesity at 3 and 5 years	Higher energy intake at 4 months was significantly associated with greater weight gain in early childhood. Higher BMI at ages 1–5 years. Increased risk of overweight at age 3 (OR = 1.46) and age 5 (OR = 1.25). These associations were observed only in formula- or mixed-fed infants, not in breastfed infants	Energy intake was based on a 1-day recall, potentially underestimating usual intake. No strong conclusions for breastfed infants due to difficulty estimating breast milk volume. Observational design: cannot establish causality. Possibility of parental reporting bias and unmeasured confounders
Multinational cross-sectional study (with retrospective feeding data) using logistic regression analyses, drawn from a large European cohort. (Papoutsou et al., 2018) [24]	Children aged 2–9 years (n = 10,808).	Introduction of solid foods before 4 months of age. Feeding data collected retrospectively from parental reports.	Introduction of solid foods at ≥4 months, including 4–6 months and 7–12 months.	Overweight and obesity measured using BMI (Cole & Lobstein criteria). Adjusted for multiple confounders (e.g., parental BMI, birth weight, smoking during pregnancy).	Early solid food introduction (before 4 months) among children who stopped exclusive breastfeeding before 4 months was associated with lower risk of overweight/obesity. Late introduction (≥7 months) was associated with increased risk of overweight/obesity among exclusively breastfed children. Best protection was found in children who were exclusively breastfed for 6 months and continued breastfeeding for ≥12 months.	Recall bias from retrospective feeding data. Feeding practice recall extended up to 8 years post-infancy. Potential residual confounding. Not a prospective cohort design
Longitudinal cohort study using U.S. Early Childhood Longitudinal Study – Birth Cohort (ECLS-B). (Salahuddin et al., 2017) [90]	4,750 children born in 2001 and followed from birth through kindergarten entry (around age 5–6)	Infant feeding practices: Timing of solid food introduction. Breastfeeding initiation and duration. Birth weight category (large-for-gestational-age vs. not)	Infants introduced to solids after 4 months (reference). Non-LGA (appropriate-for-gestational-age) children. Breastfed vs. not breastfed infants	BMI z-score trajectories from 9 months to 5 years. Overweight/obesity risk	Children introduced to solids before 4 months had higher BMI z-score trajectories. Large-for-gestational-age infants who were not breastfed and introduced to solids early had the steepest increase in BMI z-scores. Breastfeeding and delayed solids introduction appeared protective	BMI measurements were done at fixed study intervals—not continuous. Feeding practices were self-reported by parents at 9 months, possibly introducing recall bias. Study did not assess feeding quantity or quality
Observational cohort study using data from a regional child health database in Northern Ireland. (Sloan et al., 2008) [91]	Mean outcome age: 14 months (within 0–5 years range ✅)	Timing of solid food introduction. Early weaning group: before 4 months. On-time weaning group: 4 months or later	Infants weaned at or after 4 months (DoH guideline group)	Weight z-scores at 7 and 14 months. Weight gain z-score between 8 weeks and 14 months	Infants weaned before 4 months had significantly higher weight at 7 and 14 months. Early weaned infants also had more rapid weight gain between 8 weeks and 14 months. These associations remained significant after controlling for duration of breastfeeding	Feeding practices assessed retrospectively, prone to recall bias. Observational design limits ability to infer causality. Limited generalizability beyond Northern Ireland. Did not track long-term obesity or body composition beyond infancy
Cross-sectional analysis using data from the Growing Up in Australia: Longitudinal Study of Australian Children (LSAC). (Sun et al., 2016) [92]	2,423 infants aged 0–1 year at baseline	Age of solid food introduction, grouped as: before 4 months, 4–6 months, 6 months	Reference group: Infants introduced to solids between 4–6 months	BMI-for-age z-score (BMIZ) at 6–12 months	Introduction of solids before 4 months was associated with higher BMI z-scores at 6–12 months. Delayed introduction (>6 months) was not associated with BMI changes. Breastfeeding status also independently associated with BMI	Cross-sectional design; outcome and exposure measured around same time. Self-reported feeding data may have recall bias. No follow-up into later childhood to assess long-term weight effects
Cross-sectional study from a multi-country European cohort (ToyBox Study). (Usheva et al., 2021) [93]	4,578 children aged 3.5 to 5.5 years from six European countries	Age of introduction of solid foods: before 4 months, 4–5 months, ≥6 months	Children introduced to solids at 4–5 months (used as the reference group)	Weight status (overweight classification) based on BMI-for-age percentiles (WHO criteria)	No significant association was found between the timing of complementary feeding and the risk of being overweight in preschool years. Overweight status was more strongly associated with maternal BMI and education level than timing of solid food introduction	Cross-sectional design limits causal inference. Parental self-reporting of feeding practices and anthropometrics may introduce recall bias. Lack of data on portion sizes, diet quality, or feeding style. Children were older than 5 years during outcome assessment, which violates inclusion criteria from your checklist
Prospective cohort study. (Vadiveloo et al., 2019) [94]	Full-term, healthy infants followed from birth to 12 months	Timing of solid food introduction categorized as: before 4 months (early), 4–6 months (reference), 6 months (late	Infants introduced to solids at 4–6 months (reference group)	Weight-for-length z-scores (WLZ) at 12 months	Early solid food introduction (before 4 months) was significantly associated with higher WLZ at 12 months	Recall bias from self-reported feeding practices. Limited generalizability (low-income Southeastern U.S. population). Observational design limits causal conclusions
Prospective birth cohort study (Infant Feeding Practices Study II). (Wood et al., 2021) [95]	Healthy, term infants followed from birth to 12 months	Infant feeding practices in the first year, including: Breastfeeding duration. Formula use. Timing of complementary food introduction	Introduction of solids before vs. after 4 months. Feeding groups stratified by feeding type and age of solid food introduction	Rapid weight gain from birth to 12 months. Defined as increase in weight-for-age z-score >0.67 SD	Early introduction of solids (before 4 months) was significantly associated with increased risk of rapid weight gain. Breastfeeding had a protective effect against rapid weight gain. Formula-fed infants introduced early to solids were at the highest risk	Self-reported feeding data may introduce recall bias. Residual confounding from unmeasured dietary and lifestyle variables. Did not assess outcomes beyond infancy

This table summarizes key characteristics and findings of studies included in the review. It outlines the study design, population, interventions (early solid food introduction), comparators (later introduction or breastfeeding differences), measured outcomes (such as BMI, obesity prevalence, or weight-for-age z-scores), key findings, and major limitations. The studies vary in design, geographical location, and quality, but collectively assess whether introducing solid foods before four months of age contributes to increased risk of childhood obesity or accelerated growth in early life.

Study Characteristics

Seventeen studies met the inclusion criteria and were included in the final systematic review. These studies varied in design, geographic location, sample size, and analytical methods, offering a comprehensive overview of the relationship between the timing of solid food introduction and the risk of childhood obesity.

Study Design and Geographic Distribution

The included studies comprised a mix of observational designs, including prospective cohort studies (n = 9), cross-sectional studies (n = 5), secondary data analyses of cohort studies (n = 2), and a quasi-experimental study (n = 1). Data were drawn from a variety of national and multinational sources, including the United States (e.g., IFPS II, ECLS-B, and WIC), the United Kingdom (Millennium Cohort Study), Finland, Australia (LSAC), and several European cohorts (e.g., ToyBox Study, CHOP trial).

Sample Sizes and Populations

Sample sizes ranged widely across studies, from fewer than 1,000 participants to over 10,000. All studies focused on infants and young children, with age at outcome assessment ranging from early childhood (two years) to late childhood (10 years). The populations were generally representative, with several studies using nationally representative datasets.

Exposure and Outcome Measures

The main exposure assessed in all studies was the timing of solid food introduction, typically categorized as before four months versus at or after four months, with some studies using additional cutoffs such as five or six months. Most studies have assessed outcomes related to childhood obesity, using standardized measures such as body mass index (BMI), BMI z-scores, prevalence of overweight and obesity, or growth trajectories. Several studies provided more granular breakdowns of solid food introduction timing. Some reported introduction as early as two or three months, allowing a more detailed analysis of dose-response relationships between earlier feeding and obesity risk. For example, studies by Huh et al. and Ong et al. found that infants introduced to solids at ≤2 or three months had significantly higher BMI trajectories compared to those introduced after four months. This level of detail enhances understanding of the risk gradient, particularly when paired with data on breastfeeding status.

Comparators and Feeding Practices

Several studies also examined the role of infant feeding method (e.g., breastfeeding vs. formula feeding) as a potential modifier of the relationship between early introduction of solids and obesity. Some studies adjusted for or stratified by feeding practices, while others focused exclusively on certain subgroups (e.g., formula-fed or breastfed infants).

Follow-up Periods

Follow-up periods varied, with some studies assessing short-term outcomes (e.g., at two to three years of age) and others evaluating obesity risk into middle childhood (e.g., 7-10 years of age). The longer follow-up periods provided valuable insights into the lasting effects of early infant feeding practices.

Intervention Types and Materials

No interventional drug treatments or commercial feeding products were investigated in the included studies. Hence, no proprietary materials, such as specific infant formulas or nutritional supplements, were applicable or reported across the studies. Overall, the included studies provided a rich and varied dataset for analysing the association between early solid food introduction and childhood obesity risk. The diversity in study design and population enhanced the generalizability of findings, while also necessitating careful consideration of potential confounding factors and methodological heterogeneity in the synthesis.

Risk of Bias Within Studies

Across the 10 studies evaluated, no study was judged to be at low overall risk of bias, which aligns with expectations given their non-randomized designs. Five studies were rated at serious overall risk of bias [82,84,85,89-91], primarily due to inadequate control for confounding, retrospective classification of exposures, or substantial missing data. For example, Jiang and Ong did not employ advanced methods to adjust for confounding, while Gaffney had significant attrition and incomplete outcome data. The remaining five studies [94-96] were judged to be at moderate risk of bias, generally reflecting stronger designs, prospective exposure assessment, and well-defined outcomes.

Nonetheless, these studies were still subject to limitations such as residual confounding, selective reporting, or loss to follow-up. Notably, confounding was the most frequently serious-rated domain, underscoring the challenge of establishing causality in observational research on feeding practices and child weight outcomes. Despite these limitations, all studies relied on objectively measured anthropometric outcomes and were at low risk of bias in outcome measurement, lending credibility to the accuracy of reported weight and BMI data. Overall, these risks of bias assessments highlight the need for cautious interpretation of effect estimates, particularly where methodological limitations may influence internal validity.

**Figure 2:**
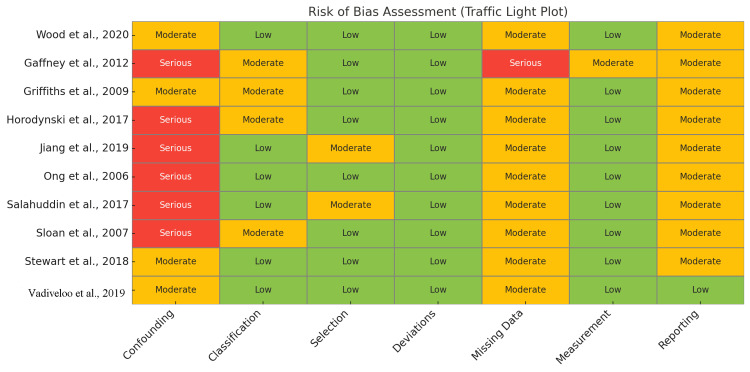
Heat map showing risk of bias judgments for the 10 included studies across seven ROBINS-I domains Risk levels are color-coded: green = low risk, orange = moderate risk, red = serious risk. No study achieved low risk across all domains; confounding was the most frequently rated domain at serious risk [82–85,89–91,94–96] ROBINS-I: Risk Of Bias In Non-randomized Studies of Interventions

Figure 2 presents a heat map summarizing the domain-level risk of bias assessments across ten non-randomized studies included in this review, as evaluated using the ROBINS-I Version 2 tool [97]. The tool assesses seven key domains: confounding, classification of interventions, selection of participants, deviations from intended interventions, missing data, measurement of outcomes, and selection of the reported result. Each cell represents the risk of bias for a given domain in a specific study, color-coded as green (low risk), orange (moderate risk), or red (serious risk). This visual format helps readers identify patterns of methodological strength and limitation across the evidence base.

Risk of Bias Assessment - Narrative Summary (AXIS Tool)

A total of six cross-sectional studies were evaluated using the AXIS tool to assess risk of bias and methodological quality [35]. Overall, the studies demonstrated moderate to high quality reporting, with several consistent strengths and a few notable limitations. Most studies had clearly stated objectives and employed appropriate study designs to investigate associations between infant feeding practices and later obesity outcomes. Sample sizes were generally large, and data were collected from nationally or regionally representative cohorts, such as the ToyBox study and ECLS-B, enhancing generalizability. However, none of the studies provided a formal sample size calculation or justification for power.

Across the studies, target populations were well defined, and data collection instruments for anthropometric and dietary variables were largely standardized. However, several studies relied on retrospective parental recall for exposure variables, such as the timing of solid food introduction, which could introduce recall bias. While statistical methods were appropriate and sufficiently detailed to enable replication, several studies did not adequately address or report on non-responders. This omission raises concerns about potential non-response bias in at least three of the studies, particularly those that excluded large numbers of participants due to missing data.

All studies presented consistent results and justified their conclusions based on the findings. Most studies acknowledged their limitations, such as potential measurement error, recall bias, or issues with generalizability, and disclosed funding sources and conflicts of interest where applicable. In summary, the AXIS assessment indicated that while the included cross-sectional studies were generally well-conducted and clearly reported, common methodological limitations include a lack of sample size justification, possible recall bias, and insufficient detail on non-responders. These factors should be considered when interpreting their findings and integrating them into the broader evidence base.

Results of Individual Studies

For each study, we present the intervention or exposure group, the comparator, the reported effect estimate with 95% confidence intervals, and the direction of the observed effect. These results reflect both adjusted and unadjusted findings, with priority given to adjusted estimates from the primary analyses. This summary facilitates the comparison of the magnitude and direction of effects across various feeding exposures and study designs.

Synthesis of Results

Non-randomized studies: This systematic review synthesized findings from ten non-randomized studies that examined associations between early infant feeding practices, including breastfeeding duration, timing of complementary food introduction, energy intake, and adherence to infant feeding guidelines and early childhood weight outcomes, such as BMI z-scores, weight-for-age z-scores (WAZ), weight-for-length z-scores (WLZ), and rapid weight gain. Across most studies, a consistent pattern emerged: shorter breastfeeding duration (particularly less than four to six months) and earlier introduction of complementary foods (before four or six months) were associated with higher weight-related outcomes by ages one to three years.

Notably, these associations were more pronounced when early solid introduction was combined with formula feeding or lower-quality diets. Studies that examined combined feeding behaviors, such as those by [94] and [96], highlighted the cumulative effect of multiple feeding exposures on growth trajectories. While effect sizes were modest (β = 0.12-0.42; ORs ~1.5-2.0), the consistency in direction across diverse contexts supports a likely positive association between early feeding practices and increased weight gain. No study reported protective effects of early weaning or short breastfeeding, and null results were limited to studies with lower-intensity interventions or limited follow-up [85].

Given the substantial heterogeneity in definitions, analytic methods, and exposure-outcome categorizations, a meta-analysis was not feasible. Studies employed different statistical metrics (e.g., regression coefficients versus odds ratios), used varying age cutoffs for breastfeeding duration and solid food introduction, and adjusted for distinct sets of covariates. This methodological diversity precluded quantitative pooling of effect estimates. As a result, synthesis relied on narrative interpretation and structured vote counting based on the direction of associations and relative effect sizes. This approach aligns with current Cochrane recommendations and ROBINS-I guidance when dealing with non-randomized evidence, where pooling across heterogeneous models may obscure rather than clarify patterns of association [97].

To complement the narrative and vote-count synthesis, Table 3 below presents a summary of effect estimates from each included study. It outlines the outcome assessed, intervention and comparator groups, effect estimates with 95% confidence intervals, and the observed direction of effect. This tabular summary facilitates cross-study comparison and illustrates the consistency of findings regarding the relationship between early feeding practices and child weight outcomes.

**Table 3:** GRADE summary of findings* ^*^[98] GRADE: Grading of Recommendations Assessment, Development and Evaluation

Outcome	No. of studies	Study design	Risk of bias	Inconsistency	Indirectness	Imprecision	Publication bias	Effect estimate	Certainty of evidence
Increased weight/BMI z-score associated with early solids or short breastfeeding	10	Non-randomized (cohort)	Serious	Moderate	No	Moderate	Unclear	OR/β range: 1.2–2.0; β = 0.12–0.42 in favor of increased weight gain	⬤⬤◯◯ Low

The GRADE evaluation identified several limitations affecting the overall certainty of the evidence. Risk of bias was rated as serious across all included studies, primarily due to concerns about residual confounding, missing outcome or exposure data, and selective reporting of results [98]. Inconsistency was present due to variability in effect sizes and exposure definitions; although most studies showed a directionally consistent association between early feeding practices and increased weight gain, the magnitude of these effects varied.

There was no serious indirectness, as the populations studied (infants in high-income countries), the exposures (infant feeding behaviors), and the outcomes (BMI z-scores, WAZ/WLZ) were directly applicable to the review objectives. Imprecision was a concern, as many studies reported wide confidence intervals and insufficient power to detect small but clinically significant differences. Lastly, publication bias could not be ruled out, as observational studies with null findings may be less likely to be published, potentially overestimating the strength of observed associations.

Cross-Sectional Studies

The six cross-sectional studies included in this review provide important but varied insights into the association between the timing of solid food introduction and the risk of childhood obesity. Below is a synthesis of their findings:

Earlier introduction and increased obesity risk: Several studies indicated a positive association between early introduction of solid foods (before four to five months) and increased risk of overweight or obesity in preschool-aged children. For example, the IDEFICS study reported that infants introduced to solids earlier had a higher likelihood of being classified as overweight or obese in early childhood.

Timing window and protective effects: The ToyBox study and the study on early childhood weight status supported the idea that delaying complementary feeding until around five to six months may offer some protection against later overweight status. These findings were consistent with current recommendations from the WHO and AAP.

Inconsistent or modest associations: Some studies, such as the Growing Up in Australia study, have found only modest or non-significant associations, highlighting the influence of confounding factors, including socioeconomic status, parental BMI, and feeding method (e.g., breastfeeding vs. formula feeding). These studies suggest that while timing plays a role, it is part of a more complex interplay of influences on child weight trajectories.

Retrospective data and confounding: All studies acknowledged limitations due to the retrospective nature of self-reporting infant feeding practices and potential residual confounding. Despite adjustments for known covariates, inconsistencies in how feeding variables were measured and categorized across studies may have contributed to variability in findings.

Heterogeneity in study design and populations: Differences in geographic regions, population characteristics, and definitions of "early" vs. "appropriate" feeding further contributed to the heterogeneity of results. For instance, studies using European cohorts (e.g., Toy Box, IDEFICS) had different cultural contexts and dietary norms compared to those using U.S. data (e.g., ECLS-B-based studies). Overall, the synthesis of cross-sectional evidence suggests a trend toward increased risk of childhood overweight or obesity with the early introduction of solid foods (before four to five months). However, the strength of this association varies depending on the study context, the measurement of exposure, and the control for confounding factors. These findings support the need for prospective studies and harmonized definitions to better understand the temporal relationship between early feeding and long-term weight outcomes.

Risk of Bias Across Studies

We conducted a qualitative assessment of the potential risk of bias across the body of evidence. Most included studies reported positive associations between early infant feeding practices (e.g., shorter breastfeeding, early solids introduction) and increased weight outcomes, suggesting a possibility of publication bias, where studies reporting null or negative findings may be underrepresented. Only one study [85] reported a null effect, and it involved a lower-intensity intervention with short-term follow-up.

Although the overall body of evidence shows consistency in the direction of associations, the tendency toward statistically significant findings and selective emphasis on positive results highlights the potential for bias across studies. These considerations further reinforce the need for cautious interpretation of results and emphasize the importance of prospective registration and full reporting in future observational research.

Discussion

Summary of Evidence

The body of evidence from the reviewed studies consistently indicates a relationship between the timing of solid food introduction and the risk of childhood overweight and obesity, although the strength and direction of associations vary by study design, population, and methodological rigor.

Early introduction (before four months) and obesity risk: Multiple studies, including large cohort and cross-sectional analyses, found that introducing solid foods before four months of age was associated with an increased risk of childhood overweight or obesity. For example, studies by Huh et al. (2011) and Moss et al. (2014) have demonstrated that the early introduction of solids significantly increases the risk of obesity by preschool age, especially among children who were never breastfed or breastfed for less than four months. This association was also supported by findings from Griffiths et al. (2009), Horodynski et al. (2017), and Sloan et al. (2008), who observed greater weight gain trajectories or higher weight-for-age scores among early-fed infants [83,84,91].

Delayed introduction (≥6 months): Delayed solid food introduction beyond six months showed mixed results. Some studies, such as those by Gaffney et al. (2012) and Papoutsou et al. (2018), suggest that introducing solids at six months or later is protective, especially when combined with prolonged breastfeeding [24,82]. However, others, such as Usheva et al. (2021), found no significant association between the introduction of solid food later in life and overweight status in preschoolers [93].

Influence of breastfeeding: The protective effect of breastfeeding was consistently highlighted across studies. A longer breastfeeding duration often moderates the risk associated with early solid food introduction. For instance, Griffiths et al. (2009) and Salahuddin et al. (2017) reported that the combination of early solids introduction and short breastfeeding duration was particularly associated with a higher BMI and an increased risk of obesity [83,90]. This was further reinforced by Differding et al. (2020), who found interactive effects of breastfeeding and complementary feeding timing on obesity outcomes [81].

Other risk modifiers: Studies such as those by Jiang et al. (2019) and Jingxiong et al. (2009) have emphasized the influence of maternal education, BMI, and socioeconomic factors on feeding practices and child weight outcomes [85,86]. The ToyBox study by Usheva et al. (2021) and Papoutsou et al. (2018) also underscored geographical and cultural variation in feeding practices and obesity prevalence [24,93].

Additionally, evidence from low- and middle-income countries (LMICs) contributes valuable context regarding cultural and socioeconomic influences on feeding practices and obesity risk. Studies conducted in China, India, and other Asian countries observed early complementary feeding practices often driven by limited maternal education, traditional beliefs, and a lack of breastfeeding support. For example, Jingxiong et al. (2009) in China and Jiang et al. (2019) highlighted that early introduction before four months was prevalent in lower-income and rural populations, correlating with higher weight-for-age z-scores and increased risk of rapid early growth [85,86]. The inclusion of such LMIC data in this review enhances the global relevance of findings and underscores the need for culturally sensitive obesity prevention strategies.

Study limitations: A recurring limitation across studies was the reliance on self-reported or retrospective feeding data, introducing recall and reporting bias. Several studies employed cross-sectional designs, which limited causal inference. Others were constrained by residual confounding, small sample sizes, or population homogeneity.

Risk of Bias Assessment

In evaluating the methodological quality of the six included cross-sectional studies using the AXIS tool, several consistent strengths and limitations were observed that are relevant for interpreting the overall findings of this systematic review [35]. The majority of studies were well-designed with clearly stated aims and utilized appropriate cross-sectional methodologies to explore associations between the timing of solid food introduction and later childhood obesity. They often relied on large, nationally or regionally representative datasets (e.g., ToyBox, ECLS-B), which enhances the external validity and generalizability of their findings.

Despite these strengths, common methodological limitations were identified. Notably, none of the studies provided a formal justification for sample size, which raises questions about the statistical power to detect associations. Additionally, several studies used retrospective parental recall to assess infant feeding practices. This introduces a potential source of recall bias that could affect the accuracy of exposure data. Another limitation observed across multiple studies was the insufficient handling or reporting of non-responders. In particular, studies that excluded large proportions of participants due to missing data (e.g., IDEFICS) may have introduced selection bias, which could affect the representativeness of the findings.

Despite these limitations, most studies used validated outcome measures (such as BMI for age percentiles) and employed appropriate statistical analyses, often adjusting for relevant confounders. They also provided transparent reporting of results and discussed study limitations in detail, further supporting the credibility of their conclusions. Overall, while the cross-sectional studies included in this review generally provide valuable insights into the relationship between early solid food introduction and obesity risk, the identified limitations, especially those related to recall bias and participant exclusions, should be taken into account when interpreting their findings and their contribution to the broader evidence base.

Implications

For clinicians and health providers: Reinforcing the importance of adhering to established feeding guidelines (e.g., avoiding solid foods before four months and promoting breastfeeding) may support healthier weight trajectories. For public health policy, programs aimed at delaying early weaning and promoting responsive feeding, particularly among formula-fed infants and low-income populations, could help reduce the risk of early obesity. For researchers, more longitudinal, culturally diverse, and rigorously designed studies are needed to clarify the optimal timing and feeding patterns.

Limitations

This systematic review has several limitations at the study, outcome, and review levels, which may affect the interpretation and generalizability of the findings.

Study-level and outcome-level limitations: Many of the included studies were cross-sectional in design, limiting the ability to establish causality between the timing of solid food introduction and subsequent obesity outcomes. Although prospective cohort studies offer stronger temporal validity, most relied on parent-reported data for infant feeding practices, which are susceptible to recall and reporting bias, especially when recall periods extended several years beyond infancy [24,91].

There was also notable variation in outcome definitions; some studies used BMI z-scores, others used weight-for-age percentiles or overweight classifications, complicating cross-study comparison. Confounding variables, such as maternal BMI, education level, and feeding practices beyond infancy, were not uniformly accounted for across studies, raising the possibility of residual confounding despite multivariate adjustments. The risk of bias assessment using the AXIS tool revealed concerns in several areas, including inadequate discussion of non-responders, insufficient justification of sample size, and inadequate description of confounder management [35]. These methodological inconsistencies may limit the internal validity of individual studies and weaken the strength of cumulative evidence.

Review-level limitations: At the review level, although a comprehensive multi-database search strategy was employed, including PubMed, Embase, Web of Science, Cochrane Library, and Google Scholar gray literature, dissertations, and non-English language publications were excluded, which may have introduced publication bias. Additionally, five full-text articles could not be retrieved, potentially omitting relevant data. While duplicate removal and screening were rigorous and conducted in duplicate, it remains possible that some eligible studies were inadvertently excluded or misclassified.

Moreover, heterogeneity in study designs, populations, exposure definitions, and outcome metrics precluded a uniform quantitative meta-analysis across all studies. As such, some synthesis was narrative, which may carry subjectivity despite being conducted systematically.

## Conclusions

Findings of this systematic review showed that introducing solid foods before four months of age is generally associated with a higher risk of childhood overweight and obesity, particularly among non-breastfed infants or those with shorter breastfeeding durations. Delaying complementary feeding until at least four months was associated with more favorable weight outcomes in most studies. While methodological limitations such as recall bias and inconsistent definitions were common, the overall trend supports adherence to current infant feeding guidelines. Future research should focus on culturally diverse longitudinal studies using standardized outcomes to better inform clinical and public health strategies.

## References

[1] (2017). Worldwide trends in body-mass index, underweight, overweight, and obesity from 1975 to 2016: a pooled analysis of 2416 population-based measurement studies in 128·9 million children, adolescents, and adults. Lancet.

[2] Di Cesare M, Sorić M, Bovet P (2019). The epidemiological burden of obesity in childhood: a worldwide epidemic requiring urgent action. BMC Med.

[3] Lauria L, Spinelli A, Buoncristiano M, Nardone P (2019). Decline of childhood overweight and obesity in Italy from 2008 to 2016: results from 5 rounds of the population-based surveillance system. BMC Public Health.

[4] Buoncristiano M, Spinelli A, Williams J (2021). Childhood overweight and obesity in Europe: changes from 2007 to 2017. Obes Rev.

[5] Anderson PM, Butcher KF, Schanzenbach DW (2019). Understanding recent trends in childhood obesity in the United States. Econ Hum Biol.

[6] Tsoi MF, Li HL, Feng Q, Cheung CL, Cheung TT, Cheung BM (2022). Prevalence of childhood obesity in the United States in 1999-2018: a 20-year analysis. Obes Facts.

[7] Daepp MI, Gortmaker SL, Wang YC, Long MW, Kenney EL (2019). WIC food package changes: trends in childhood obesity prevalence. Pediatrics.

[8] Bendor CD, Bardugo A, Pinhas-Hamiel O, Afek A, Twig G (2020). Cardiovascular morbidity, diabetes and cancer risk among children and adolescents with severe obesity. Cardiovasc Diabetol.

[9] Caprio S, Santoro N, Weiss R (2020). Childhood obesity and the associated rise in cardiometabolic complications. Nat Metab.

[10] Smith JD, Fu E, Kobayashi M (2020). Prevention and management of childhood obesity and its psychological and health comorbidities. Annu Rev Clin Psychol.

[11] Must A, Hollander SA, Economos CD (2006). Childhood obesity: a growing public health concern. Expert Rev Endocrinol Metab.

[12] Koletzko B, Fishbein M, Lee WS, Moreno L, Mouane N, Mouzaki M, Verduci E (2020). Prevention of childhood obesity: a position paper of the Global Federation of International Societies of Paediatric Gastroenterology, Hepatology and Nutrition (FISPGHAN). J Pediatr Gastroenterol Nutr.

[13] Lee EY, Yoon KH (2018). Epidemic obesity in children and adolescents: risk factors and prevention. Front Med.

[14] Roth CL, Jain V (2018). Rising obesity in children: a serious public health concern. Indian J Pediatr.

[15] WHO WHO (2025). WHO: exclusive breastfeeding for optimal growth, development and health of infants. https://www.who.int/tools/elena/interventions/exclusive-breastfeeding.

[16] Meek JY, Noble L (2022). Policy statement: breastfeeding and the use of human milk. Pediatrics.

[17] Fewtrell M, Bronsky J, Campoy C (2017). Complementary feeding: a position paper by the European Society for Paediatric Gastroenterology, Hepatology, and Nutrition (ESPGHAN) Committee on Nutrition. J Pediatr Gastroenterol Nutr.

[18] Appleton J, Russell CG, Laws R, Fowler C, Campbell K, Denney-Wilson E (2018). Infant formula feeding practices associated with rapid weight gain: a systematic review. Matern Child Nutr.

[19] Clayton PK, Putnick DL, Trees IR, Ghassabian A, Tyris JN, Lin TC, Yeung EH (2024). Early infant feeding practices and associations with growth in childhood. Nutrients.

[20] Dharod JM, Black MM, McElhenny K, Labban JD, DeJesus JM (2024). Es Niño o Niña?: Gender differences in feeding practices and obesity risk among Latino infants. Curr Dev Nutr.

[21] Koletzko B, von Kries R, Closa R (2009). Can infant feeding choices modulate later obesity risk?. Am J Clin Nutr.

[22] Owen CG, Martin RM, Whincup PH, Smith GD, Cook DG (2005). Effect of infant feeding on the risk of obesity across the life course: a quantitative review of published evidence. Pediatrics.

[23] Savage JS, Hohman EE, Marini ME, Shelly A, Paul IM, Birch LL (2018). INSIGHT responsive parenting intervention and infant feeding practices: randomized clinical trial. Int J Behav Nutr Phys Act.

[24] Papoutsou S, Savva SC, Hunsberger M (2018). Timing of solid food introduction and association with later childhood overweight and obesity: the IDEFICS study. Matern Child Nutr.

[25] Moorcroft KE, Marshall JL, McCormick FM (2011). Association between timing of introducing solid foods and obesity in infancy and childhood: a systematic review. Matern Child Nutr.

[26] Seach KA, Dharmage SC, Lowe AJ, Dixon JB (2010). Delayed introduction of solid feeding reduces child overweight and obesity at 10 years. Int J Obes (Lond).

[27] Huh SY, Rifas-Shiman SL, Taveras EM, Oken E, Gillman MW (2011). Timing of solid food introduction and risk of obesity in preschool-aged children. Pediatrics.

[28] Cohen CC, Harrall KK, Hu H, Glueck DH, Perng W, Shankar K, Dabelea D (2024). Associations of infant feeding practices with abdominal and hepatic fat measures in childhood in the longitudinal Healthy Start Study. Am J Clin Nutr.

[29] Vehapoglu A, Yazıcı M, Demir AD, Turkmen S, Nursoy M, Ozkaya E (2014). Early infant feeding practice and childhood obesity: the relation of breast-feeding and timing of solid food introduction with childhood obesity. J Pediatr Endocrinol Metab.

[30] Dalrymple K, Gallagher S, Flynn A, Poston L (2024). Infant feeding practices: an analysis of sociodemographic characteristics and dietary patterns in early life. Proc Nutr Soc.

[31] Barrera CM, Perrine CG, Li R, Scanlon KS (2016). Age at introduction to solid foods and child obesity at 6 years. Child Obes.

[32] (2025). PRISMA 2020 statement. PRISMA statement. https://www.prisma-statement.org/prisma-2020.

[33] (2025). Rayyan: AI-powered systematic review management platform. https://www.rayyan.ai/.

[34] (2025). Risk of bias tools - ROBINS-I tool. https://www.riskofbias.info/welcome/home.

[35] (2025). AXIS tool. Latitudes Network. https://www.latitudes-network.org/tool/axis-tool/.

[36] (2025). Ottawa Hospital Research Institute. https://www.ohri.ca/programs/clinical_epidemiology/oxford.asp.

[37] O'Donovan SM, Murray DM, Hourihane JO, Kenny LC, Irvine AD, Kiely M (2015). Adherence with early infant feeding and complementary feeding guidelines in the Cork BASELINE Birth Cohort Study. Public Health Nutr.

[38] Vail B, Prentice P, Dunger DB, Hughes IA, Acerini CL, Ong KK (2015). Age at weaning and infant growth: primary analysis and systematic review. J Pediatr.

[39] Kim J, Peterson KE (2008). Association of infant child care with infant feeding practices and weight gain among US infants. Arch Pediatr Adolesc Med.

[40] Neri D, Oliveira FL, Carvalho AM, Somarriba GA, Scott GB, Miller TL (2017). Associations between dietary intake before 6 months of age and rapid weight gain among HIV-exposed uninfected infants. J Pediatr Gastroenterol Nutr.

[41] Dharod JM, Hernandez M, Labban JD (2023). Associations between early introduction to complementary foods, subsequent cereal-added bottle feeding and daily macronutrient intake among infants. Appetite.

[42] de Beer M, Vrijkotte TG, Fall CH, van Eijsden M, Osmond C, Gemke RJ (2015). Associations of infant feeding and timing of linear growth and relative weight gain during early life with childhood body composition. Int J Obes (Lond).

[43] Grote V, Theurich M, Luque V, Gruszfeld D, Verduci E, Xhonneux A, Koletzko B (2018). Complementary feeding, infant growth, and obesity risk: timing, composition, and mode of feeding. Nestle Nutr Inst Workshop Ser.

[44] Gingras V, Aris IM, Rifas-Shiman SL, Switkowski KM, Oken E, Hivert MF (2019). Timing of complementary feeding introduction and adiposity throughout childhood. Pediatrics.

[45] Chivers P, Hands B, Parker H, Bulsara M, Beilin LJ, Kendall GE, Oddy WH (2010). Body mass index, adiposity rebound and early feeding in a longitudinal cohort (Raine Study). Int J Obes (Lond).

[46] Bonuck K, Avraham SB, Lo Y, Kahn R, Hyden C (2014). Bottle-weaning intervention and toddler overweight. J Pediatr.

[47] Fewtrell MS (2016). Can optimal complementary feeding improve later health and development?. Nestle Nutr Inst Workshop Ser.

[48] Emmett PM, Jones LR (2014). Diet and growth in infancy: relationship to socioeconomic background and to health and development in the Avon Longitudinal Study of Parents and Children. Nutr Rev.

[49] Bolton KA, Kremer P, Hesketh KD, Laws R, Kuswara K, Campbell KJ (2018). Differences in infant feeding practices between Chinese-born and Australian-born mothers living in Australia: a cross-sectional study. BMC Pediatr.

[50] Noble S, Emmett P (2006). Differences in weaning practice, food and nutrient intake between breast- and formula-fed 4-month-old infants in England. J Hum Nutr Diet.

[51] Fangupo LJ (2016). Does a ‘Baby-Led’ Approach to Complementary Feeding Alter the Risk of Choking and Growth Faltering in Infants Aged 0-12 Months?. https://ourarchive.otago.ac.nz/esploro/outputs/graduate/Does-a-baby-led-approach-to-complementary/9926479958401891#files_and_links_(1).

[52] Wen LM, De Domenico M, Elliott D, Bindon J, Rissel C (2009). Evaluation of a feasibility study addressing risk factors for childhood obesity through home visits. J Paediatr Child Health.

[53] Abraham EC, Godwin J, Sherriff A, Armstrong J (2012). Infant feeding in relation to eating patterns in the second year of life and weight status in the fourth year. Public Health Nutr.

[54] Camier A, Cissé AH, Heude B (2024). Infant feeding practices and body mass index up to 7.5 years in the French nationwide ELFE study. Pediatr Obes.

[55] Aris IM, Bernard JY, Chen LW (2017). Modifiable risk factors in the first 1000 days for subsequent risk of childhood overweight in an Asian cohort: significance of parental overweight status. Int J Obes (Lond).

[56] Yarnoff B, Allaire B, Detzel P (2014). Mother, infant, and household factors associated with the type of food infants receive in developing countries. Front Pediatr.

[57] Taylor RW, Conlon CA, Beck KL (2021). Nutritional implications of baby-led weaning and baby food pouches as novel methods of infant feeding: protocol for an observational study. JMIR Res Protoc.

[58] Razak NA, Muniandy ND (2019). Nutritional profile of commercial infant and toddler food products available in Klang Valley. Healthscope.

[59] Narain B, Dubash PJ (1976). Nutritional requirements of infants and need for supplementing milk diet with infant weaning foods. Indian J Pediatr.

[60] Metwally AM, Sallam SF, Mawla MA (2022). Promoting weaning practices and growth of Egyptian infants by using communication for behavioral development approach. BMC Pediatr.

[61] Nansel TR, Channell-Doig A, Lipsky LM, Burger K, Shearrer G, Siega-Riz AM, Ma Y (2024). Prospective associations of infant food exposures and appetitive traits with early childhood diet quality. Int J Behav Nutr Phys Act.

[62] Milani GP, Edefonti V, De Cosmi V (2023). Protein and growth during the first year of life: a systematic review and meta-analysis. Pediatr Res.

[63] Günther AL, Buyken AE, Kroke A (2007). Protein intake during the period of complementary feeding and early childhood and the association with body mass index and percentage body fat at 7 y of age. Am J Clin Nutr.

[64] Kumaran K, Krishnaveni GV, Suryanarayana KG (2021). Protocol for a cluster randomised trial evaluating a multifaceted intervention starting preconceptionally-Early Interventions to Support Trajectories for Healthy Life in India (EINSTEIN): a Healthy Life Trajectories Initiative (HeLTI) Study. BMJ Open.

[65] Lande B, Andersen LF, Henriksen T (2005). Relations between high ponderal index at birth, feeding practices and body mass index in infancy. Eur J Clin Nutr.

[66] Thorisdottir AV, Gunnarsdottir I, Thorsdottir I (2013). Revised infant dietary recommendations: the impact of maternal education and other parental factors on adherence rates in Iceland. Acta Paediatr.

[67] Schäfer T, Bauer CP, Beyer K (2014). S3-Guideline on allergy prevention: 2014 update: guideline of the German Society for Allergology and Clinical Immunology (DGAKI) and the German Society for Pediatric and Adolescent Medicine (DGKJ). Allergo J Int.

[68] Murray RD (2017). Savoring sweet: sugars in infant and toddler feeding. Ann Nutr Metab.

[69] Michaelsen KF, Larnkjaer A, Lauritzen L, Mølgaard C (2010). Science base of complementary feeding practice in infancy. Curr Opin Clin Nutr Metab Care.

[70] Gibbs BG, Forste R (2014). Socioeconomic status, infant feeding practices and early childhood obesity. Pediatr Obes.

[71] Davis KE, Klingenberg A, Massey-Stokes M, Habiba N, Gautam R, Warren C, Yeatts P (2023). The Baby Bites Text Messaging Project with randomized controlled trial: texting to improve infant feeding practices. Mhealth.

[72] Askie LM, Baur LA, Campbell K (2010). The Early Prevention of Obesity in CHildren (EPOCH) Collaboration--an individual patient data prospective meta-analysis. BMC Public Health.

[73] Daniels LA, Magarey A, Battistutta D, Nicholson JM, Farrell A, Davidson G, Cleghorn G (2009). The NOURISH randomised control trial: Positive feeding practices and food preferences in early childhood - a primary prevention program for childhood obesity. BMC Public Health.

[74] de Souza GR, Ribeiro-Silva RC, Felisbino-Mendes MS (2023). Time trends and social inequalities in infant and young child feeding practices: national estimates from Brazil's Food and Nutrition Surveillance System, 2008-2019. Public Health Nutr.

[75] Sithamparapillai K, Samaranayake D, Wickramasinghe VP (2022). Timing and pattern of growth faltering in children up-to 18 months of age and the associated feeding practices in an urban setting of Sri Lanka. BMC Pediatr.

[76] Ierodiakonou D, Garcia-Larsen V, Logan A (2016). Timing of allergenic food introduction to the infant diet and risk of allergic or autoimmune disease: a systematic review and meta-analysis. JAMA.

[77] Helle C, Hillesund ER, Øverby NC (2018). Timing of complementary feeding and associations with maternal and infant characteristics: a Norwegian cross-sectional study. PLoS One.

[78] Martin MA, Glass DJ (2025). Timing of complementary feeding and infant growth trajectories in prospective cohort studies: a systematized review and analysis of socioecological variation. Ecol Food Nutr.

[79] Studer-Perez E, Musher-Eizenman D (2023). To feed or let eat! A scale of independence, exploration, and family to measure baby-led weaning as a complementary feeding approach. J Hum Nutr Diet.

[80] Goyena E, Maniego LV, Cristobal AG (2023). Validation of selected 2021 infant and young child feeding indicators for appropriate complementary feeding in relation to dietary adequacy and anthropometric status. Mal J Nutr.

[81] Differding MK, Doyon M, Bouchard L (2020). Potential interaction between timing of infant complementary feeding and breastfeeding duration in determination of early childhood gut microbiota composition and BMI. Pediatr Obes.

[82] Gaffney KF, Kitsantas P, Cheema J (2012). Clinical practice guidelines for feeding behaviors and weight-for-age at 12 months: a secondary analysis of the Infant Feeding Practices Study II. Worldviews Evid Based Nurs.

[83] Griffiths LJ, Smeeth L, Hawkins SS, Cole TJ, Dezateux C (2009). Effects of infant feeding practice on weight gain from birth to 3 years. Arch Dis Child.

[84] Horodynski MA, Pierce SJ, Reyes-Gastelum D, Olson B, Shattuck M (2017). Feeding practices and infant growth: quantifying the effects of breastfeeding termination and complementary food introduction on BMI z-score growth velocity through growth curve models. Child Obes.

[85] Jiang H, Li M, Wen LM, Baur L, He G, Ma X, Qian X (2019). A community-based short message service intervention to improve mothers' feeding practices for obesity prevention: quasi-experimental study. JMIR Mhealth Uhealth.

[86] Jingxiong J, Rosenqvist U, Huishan W, Koletzko B, Guangli L, Jing H, Greiner T (2009). Relationship of parental characteristics and feeding practices to overweight in infants and young children in Beijing, China. Public Health Nutr.

[87] Moschonis G, de Lauzon-Guillain B, Jones L (2017). The effect of early feeding practices on growth indices and obesity at preschool children from four European countries and UK schoolchildren and adolescents. Eur J Pediatr.

[88] Moss BG, Yeaton WH (2014). Early childhood healthy and obese weight status: potentially protective benefits of breastfeeding and delaying solid foods. Matern Child Health J.

[89] Ong KK, Emmett PM, Noble S, Ness A, Dunger DB (2006). Dietary energy intake at the age of 4 months predicts postnatal weight gain and childhood body mass index. Pediatrics.

[90] Salahuddin M, Pérez A, Ranjit N, Hoelscher DM, Kelder SH (2017). The associations of large-for-gestational-age and infant feeding practices with children's body mass index z-score trajectories: the Early Childhood Longitudinal Study, Birth Cohort. Clin Obes.

[91] Sloan S, Gildea A, Stewart M, Sneddon H, Iwaniec D (2008). Early weaning is related to weight and rate of weight gain in infancy. Child Care Health Dev.

[92] Sun Z, Xu W, Huang S, Chen Y, Guo X, Shi Z (2016). Dual-source computed tomography evaluation of children with congenital pulmonary valve stenosis. Iran J Radiol.

[93] Usheva N, Galcheva S, Cardon G (2021). Complementary feeding and overweight in European preschoolers: the ToyBox-study. Nutrients.

[94] Vadiveloo M, Tovar A, Østbye T, Benjamin-Neelon SE (2019). Associations between timing and quality of solid food introduction with infant weight-for-length z-scores at 12 months: findings from the Nurture cohort. Appetite.

[95] Wood CT, Witt WP, Skinner AC (2020). Effects of breastfeeding, formula feeding, and complementary feeding on rapid weight gain in the first year of life. Acad Pediatr.

[96] Stewart CJ, Ajami NJ, O'Brien JL (2018). Temporal development of the gut microbiome in early childhood from the TEDDY study. Nature.

[97] (2025). Risk of bias tools - ROBINS-I V2 tool. https://www.riskofbias.info/welcome/robins-i-v2.

[98] Guyatt GH, Oxman AD, Vist GE, Kunz R, Falck-Ytter Y, Alonso-Coello P, Schünemann HJ (2008). GRADE: an emerging consensus on rating quality of evidence and strength of recommendations. BMJ.

